# Prevalence of hypothermia prior to trauma center arrival and associated mortality in pediatric traumatic brain injury patients

**DOI:** 10.1007/s00383-026-06650-w

**Published:** 2026-09-22

**Authors:** Teresa M. Bell, Jodi Raymond, Andrea N. Davis, Mathew P. Landman, Laurie Ackerman

**Affiliations:** 1 Department of Surgery, University of Utah, Salt Lake City, UT USA; 2 Riley Hospital for Children at Indiana University Health, Indianapolis, IN USA; 3 Department of Surgery, Indiana University, Indianapolis, IN USA; 4 Neurosurgery, Indiana University, Indianapolis, IN USA; 5 IU School of Medicine, Trauma Medical Director at Riley Hospital for Children at IU Health, 340 West 10th Street Fairbanks Hall, Suite 6200, Indianapolis, IN 46202-3082 USA

**Keywords:** Hypothermia, Traumatic brain injury (TBI), Pediatric trauma, Neurosurgery, Care transitions, Emergency medical services (EMS)

## Abstract

**Objective:**

Hypothermia is a component of the trauma “lethal triad” but often goes untreated. The prevalence of prehospital hypothermia in pediatric traumatic brain injury (TBI) patients is unknown. We aimed to examine how frequently hypothermia occurred during prehospital care by emergency medical services (EMS), at outside transferring hospitals (OSH), and during interfacility transport (IF) prior to trauma center arrival.

**Methods:**

This was a retrospective cohort study of patients < 18 years old treated for TBI and admitted to the intensive care unit at a level 1 pediatric trauma center. Body temperature was categorized as normal (≥ 36 °C), mild (34–36 °C), moderate (32–34 °C), or severe (< 32 °C) hypothermia using EMS, OSH, IF, and arrival ED measurements.

**Results:**

Among 171 patients, 40.4% experienced prehospital hypothermia. Mortality was higher in hypothermic patients (49.3%) compared with normothermic patients (15.7%; *p* < 0.001). Hypothermia was more frequent in children < 2 years (55.1%), non-accidental trauma (42%), and transfers (66.7%). Temperature was undocumented in 84.6% of EMS, 28.1% of IF, and 33.3% of OSH encounters. Patients without documented temperatures often arrived at the trauma center with temperatures below 34 °C.

**Conclusions:**

Prehospital hypothermia is common and strongly associated with mortality in pediatric TBI, yet frequently unassessed. Routine prehospital temperature monitoring should be prioritized.

## Introduction

 Hypothermia is considered part of the “Lethal Triad” in trauma patients, although it often goes undiagnosed [1]. Hypothermia in trauma patients has been associated with increased mortality rates, intensive care unit admission rates, and poor outcomes [2–4]. Hypothermia at admission has been associated with significantly higher mortality rates in trauma patients overall and specifically in patients with traumatic brain injury (TBI) [5].

The frequency of hypothermia in injured patients is often underreported, although one study found that 29% of trauma victims were hypothermic upon EMS arrival on scene [6]. Less is known about the occurrence of unintentional hypothermia in the prehospital setting among pediatric TBI patients, who are particularly susceptible to developing low body temperatures that require clinical intervention [7]. Studies have reported varying rates of hypothermia upon arrival at trauma centers, ranging from 0.2% to 47% [6, 8–10]. Although body temperature is considered a critical vital sign and can be measured quickly and noninvasively in field settings, it is often not collected and is not a standard national emergency medical services (EMS) data element [11, 12]. The lack of assessment and undertreatment of hypothermia in the initial management of severe trauma has been identified as a critical gap in prehospital care, suggesting that increased attention and focus on addressing hypothermia in prehospital settings could improve patient outcomes [6, 11].

To our knowledge, no studies have examined hypothermia across prehospital care transitions in pediatric head trauma patients and its downstream effect on trauma center outcomes. To address this gap, this study aims to examine the prevalence of hypothermia prior to trauma center arrival in pediatric TBI patients requiring intensive care. We evaluated patient temperature during prehospital EMS care, at outside hospitals (OSH) prior to transfer, and during interfacility (IF) transport to determine if hypothermia was associated with worse outcomes in the definitive care setting, including death and discharge to inpatient rehabilitation facilities.

## Methods

### Data source

Following institutional review board (IRB) approval and a waiver of consent from the Indiana University IRB, a retrospective chart review was conducted using the electronic health record (EHR) system at Riley Hospital for Children at Indiana University Health, a pediatric Level 1 trauma center. The review included patients treated for TBI between January 1, 2015, and December 31, 2018. Inclusion criteria were all patients 18 years of age or younger admitted to the pediatric intensive care unit with a head injury based on Abbreviated Injury Scale (AIS) coding, and patients with a Glasgow Coma Scale (GCS) score less than or equal to 8 in the emergency department. EMS, electronic health records, and trauma registry data were collected and managed using the REDCap electronic data capture tool hosted by Indiana University.

Data collected during EHR chart review included demographic variables such as age, sex, race, ethnicity, insurance type, home zip code, and comorbidities. Injury characteristics, including mechanism of injury, intent, date and time, AIS scores, and injury severity scores (ISS) were obtained from the trauma registry. Variables collected from EMS, OSH, and IF records included type of transport, GCS, vital signs and timestamps, blood gas measurements, blood gas values, lab values, medications, and complications, including seizure.

### Variables

The primary outcome of the study was in-hospital death or discharge to an inpatient rehabilitation facility. The independent variable of interest, hypothermia (< 36 °C), was determined using vital measurements taken during prehospital EMS care, at outside transferring hospitals (OSH), during interfacility transport from the outside hospital to the trauma center (IF), and upon ED arrival. All temperature measurements were checked for validity and converted to Celsius. Measurements closest in time to EMS scene arrival and OSH arrival were used if multiple temperature measurements were available. The lowest temperature recorded during IF was collected along with timestamps. If vitals were missing for a patient who did receive care in a setting, it was confirmed in the patient’s chart narrative that no temperature was recorded, and only then was the value coded as “not collected.” If patients did not receive care in a specific setting, temperature was recorded as “not applicable. For example, patients arriving by private vehicle would have an EMS temperature of “not applicable” rather than missing. Similarly, patients who were not transferred and presented directly to the trauma center would have “not applicable” listed for OSH and IF temperatures. Hypothermia severity was further categorized as mild (34–35.9°C), moderate (32–33.9°C), and severe (< 32 °C). Binary hypothermia variables and categorical variables for the presence and severity of hypothermia were computed for each stage of care. Composite variables indicating whether hypothermia had been present at any stage prior to trauma center arrival and severity category based on the lowest temperature recorded prior to trauma center arrival were also examined.

### Statistical analysis

Descriptive statistics, including means and frequencies, were calculated for the cohort’s demographic, clinical, and outcome characteristics. The proportion of patients experiencing hypothermia in each care setting is also presented. We used the chi-square and Fisher’s exact tests to examine the association between pre-trauma center arrival hypothermia and trauma center outcomes. Fisher-Freeman-Halton exact tests were used to examine associations when expected cell counts were below 5, and categorical variables had more than two levels. We also performed a subanalysis on patients 0 to 2 years of age to determine whether mortality was more often observed after hypothermia in younger children. Lastly, we examined whether hypothermia at earlier stages of care was associated with hypothermia severity progression across care transitions. Differences in the proportion of hypothermia cases by month were also assessed to examine whether season was associated with low body temperature. All tests were two-sided, and alpha was set at 0.05.

## Results

### Cohort demographics and injury characteristics

Our study included 171 patients who were predominantly male (*n* = 106, 62%), white (*n* = 135, 78.3%), and publicly insured (*n* = 102, 59.6%). Patient temperatures were not recorded for 84.9% of EMS transports (124 of 146 transports from scene), 28.9% of IF transports (28 of 97), and 34% of OSH transfers (33 of 97). A total of 25 patients arrived at the hospital by private vehicle rather than EMS transport and 74 patients presented directly to the trauma center. Moderate or severe hypothermia on arrival to the trauma center was most often present in patients missing temperature data (15.7% missing EMS, 18.8% missing OSH, and 14.8% missing IF had moderate to severe hypothermia upon trauma center arrival). Over half of the patients were transferred from an OSH (*n* = 97, 56.7%) and had injury severity scores over 15 (*n* = 126, 73.6%). Approximately a third of patients were two or younger (*n* = 58, 33.9%). The most common mechanism of injury was motor vehicle crash (*n* = 77, 45%), followed by non-accidental trauma (*n* = 39, 22.8%), and other (*n* = 31, 18.1%). The majority of patients had multiple injuries with at least one other body region injured in addition to their head (*n* = 132, 77.2%). The most frequent maximum non-head AIS score was 2 (*n* = 46, 26.9%), and nearly one-third of patients had non-head AIS scores of 3 or greater (*n* = 50, 29.2%). (Table 1)

### Characteristics of patients with hypothermia

Sixty-nine patients (40.4%) experienced hypothermia prior to trauma center arrival in at least one stage of care. Hypothermia was more common in patients under the age of 2 (55.1% vs. 21.7% in ages 3–10 and 23.2% in those 11 and older) and in patients who were transferred from an OSH (66.7% vs. 33.3% in non-transfer patients). Non-accidental trauma and motor vehicle crashes were the most common mechanisms of injury for hypothermic patients (42% and 30.4%, respectively). Lower GCS and higher ISS scores were associated with hypothermia. Sex, race, insurance type, multiple injuries, and maximum non-head AIS were not associated with hypothermia prior to trauma center arrival. (Table 1)

Hypothermia occurred at a similar frequency across all months in the study (*p* = 0.168, results not shown due to table length).

### Discharge outcomes

Death occurred in 49.3% of patients with hypothermia (*n* = 34) compared to 15.7% of patients who experienced no hypothermia prior to trauma center arrival (*n* = 16). Excluding patients who died, significantly fewer patients who experienced hypothermia required inpatient rehabilitation (37.1%) than normothermic patients (58.1%). We found that hypothermia severity was also associated with poor patient outcomes, with death occurring in 80% of patients with severe hypothermia (*n* = 8), 73.3% in those with moderate hypothermia (*n* = 11), and 34.1% in mild hypothermia (*n* = 15) as opposed to 15.7% (*n* = 16) with no hypothermia (*p* < 0.001). Discharge to inpatient rehabilitation was most common in normothermic patients (49% compared to 22.7% with mild hypothermia, 13.3% moderate, and 10% severe, *n* = 61 total discharged to inpatient rehabilitation). (Table 2)

**Table 1:** Cohort descriptives (*n* = 171)

		No Hypothermia		Hypothermia		*p* value
Age	2 and Younger	20	19.6%	38	55.1%	< 0.001
	3–10	45	44.1%	15	21.7%	
	11 and Older	37	36.3%	16	23.2%	
Sex	Female	42	41.2%	23	33.3%	0.3
	Male	60	58.8%	46	66.7%	
Race	White	77	77.8%	53	79.1%	0.504
	Black	20	20.2%	14	20.9%	
	Asian	2	2.0%	0	0.0%	
Insurance type	Public	58	56.9%	44	63.8%	0.091
	Private	41	40.2%	19	27.5%	
	Self-pay	3	2.9%	6	8.7%	
Transfer	No	51	50.0%	23	33.3%	0.031
	Yes	51	50.0%	46	66.7%	
Mechanism	Bicycle	4	3.9%	2	2.9%	< 0.001
	Fall	10	9.8%	8	11.6%	
	MVC	56	54.9%	21	30.4%	
	NAT	10	9.8%	29	42.0%	
	Other	22	21.6%	9	13.0%	
First GCS	< 8	57	55.9%	57	82.6%	< 0.001
	9–13	30	29.4%	6	8.7%	
	14–15	15	14.7%	6	8.7%	
ED Arrival GCS	< 8	94	92.2%	69	100.0%	0.059
	9–13	7	6.9%	0	0.0%	
	14–15	1	1.0%	0	0.0%	
ISS	< 10	16	15.7%	5	7.2%	0.008
	11–15	12	11.8%	12	17.4%	
	16–25	29	28.4%	8	11.6%	
	> 25	45	44.1%	44	63.8%	
Multiple injury	No	26	25.5%	13	18.8%	0.309
	Yes	76	74.5%	56	81.2%	
Maximum non-Head AIS Score	0	26	25.5%	12	17.6%	0.368
	1	16	15.7%	20	29.4%	
	2	28	27.5%	18	26.5%	
	3	20	19.6%	11	16.2%	
	4	9	8.8%	6	8.8%	
	5	3	2.9%	1	1.5%	

**Table 2:** Hypothermia severity prior to trauma center arrival and discharge outcomes

Hypothermia severity	Discharge disposition						
	Routine		Inpatient Rehab		Died		*p* value
Normal (36+)	36	35.3%	50	49.0%	16	15.7%	< 0.001
Mild (34–36)	19	43.2%	10	22.7%	15	34.1%	
Moderate (32–34)	2	13.3%	2	13.3%	11	73.3%	
Severe (< 32)	1	10.0%	1	10.0%	8	80.0%	

### Children 2 and younger

Only one-third of children 2 years and younger maintained normal temperatures across all care settings prior to trauma center arrival (*n* = 20), compared to 72.6% of children older than 2 years (*n* = 82). Younger children more often had greater severity of hypothermia, with 34.5% experiencing mild hypothermia (*n* = 20), 20.7% moderate (*n* = 12), and 10.3% severe hypothermia (*n* = 6), compared to 21.2% (*n* = 24), 2.7% (*n* = 3), and 3.5% (*n* = 6) in children over 2, respectively (*p* < 0.001). Young children with more severe hypothermia had worse outcomes, with 100% of children with severe hypothermia dying prior to hospital discharge (*n* = 6), as well as 66.7% with moderate hypothermia (*n* = 8) and 40% with mild hypothermia (*n* = 8). Of the younger children who were discharged to inpatient rehabilitation, 15.4% had mild hypothermia (*n* = 2) and 15.4% had moderate hypothermia (*n* = 2). (Table 3)

**Table 3:** Outcomes of children age 2 and younger

Temperature	Discharge disposition						
	Routine		Inpatient Rehab		Died		*p* value
Normal	7	36.8%	9	69.2%	4	15.4%	0.002
Mild Hypothermia	10	52.6%	2	15.4%	8	30.8%	
Moderate Hypothermia	2	10.5%	2	15.4%	8	30.8%	
Severe Hypothermia	0	0.0%	0	0.0%	6	23.1%	

### Progression of hypothermia across care transitions

**Fig. 1:**
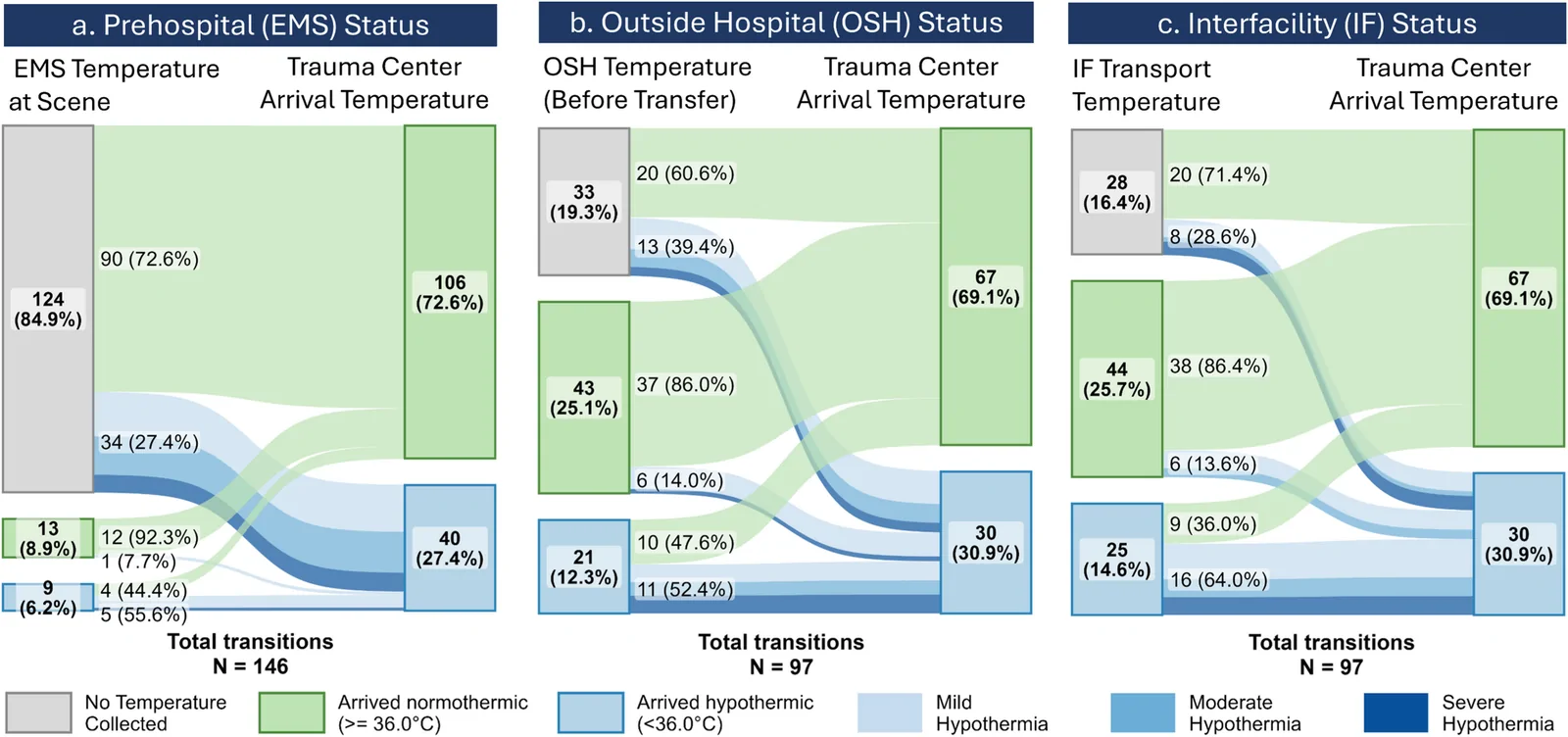
Temperature across EMS transport from scene, at outside transferring hospital, and during interfacility transport prior to trauma center arrival (*N* = 171). Panel **a** Excludes private-vehicle arrivals (no EMS transport/temperature status; *n* = 25)

We examined temperature in EMS, OSH, and IF care phases, along with the severity of hypothermia at trauma center arrival. We found that hypothermia at the OSH and during IF transport was associated with trauma center arrival temperature. 60% of patients recorded as having mild hypothermia in the OSH had normal temperatures upon arrival at the trauma center (*n* = 9). However, the majority of patients with moderate and severe hypothermia at the OSH were still hypothermic on trauma center arrival (*n* = 5, 83.3%). Among patients who had no temperature collected at the OSH, 40.7% (*n* = 13) had hypothermia upon trauma center arrival. During IF transport, 53.3% of patients with mild hypothermia reached normal body temperature by ED arrival at the trauma center. However, the majority of patients with moderate and severe hypothermia did not improve, or their temperature worsened by the time of ED arrival. Approximately 30% of patients who had no IF transport temperature collected had hypothermia at ED arrival (*n* = 8). (Fig. 1)

## Discussion

Any documented episode of hypothermia during care transitions prior to trauma center arrival was associated with increased mortality in this cohort of pediatric TBI patients. Although death was most common in patients with more severe hypothermia, even minor hypothermia was associated with increased mortality. Hypothermia occurred more frequently in children under the age of 2, and this age group was often the most likely to present with lower temperatures. In fact, the majority of patients under the age of 2 years arrived at the trauma center hypothermic. Additionally, patients transferred from another facility were more likely to experience hypothermia prior to presenting at the trauma center. Often, transfer patients had progressively lower temperatures as they transitioned between each stage of care. Transport patients with mild hypothermia were often able to recover by the time they reached the trauma center. However, in cases where hypothermia was moderate or severe, body temperatures were slower to recover.

These results are in line with previous studies in adult patients, in which arrival hypothermia was associated with increased mortality [13, 14] and poorer outcomes [15, 16]. The association between temperature on arrival and in-hospital mortality has also been found in the general pediatric trauma population. While a number of studies have examined the use of therapeutic hypothermia in children [17–19]. Fewer studies have examined the effects of unintentional hypothermia on outcomes in pediatric TBI patients [20]. Interestingly, our study found that patients who survived but were hypothermic upon arrival to the trauma center were less likely to be discharged to inpatient rehabilitation than normothermic patients. However, this effect is possibly due to survival bias, as significantly more deaths occurred in the hypothermic group.

The findings of the present study highlight several important considerations for the early care of pediatric patients with TBI. Regarding prehospital hypothermia, our results indicate that patient temperature often goes unassessed during EMS transport, and over a quarter of children without EMS temperatures recorded arrive at the trauma center with hypothermia. Collection of this important vital sign should be routine when transporting trauma patients so that warming interventions can be initiated before reaching more severe stages of hypothermia.

Interventions to maintain a stable temperature at the transferring hospitals and during transport are also important, as low body temperature can delay life-saving interventions upon arrival at the trauma center. The EPIC4Kids study reported potential benefits of standardized, prehospital management of pediatric TBI, with guideline implementation associated with improved survival among children with severe TBI [21]. However, these guidelines were primarily focused on treating hypoxia, hypotension, and hyperventilation in the prehospital setting, as opposed to hypothermia [22]. Younger children with head injuries, who are smaller and less able to maintain stable body temperatures, should be considered particularly at risk for developing hypothermia that requires rapid intervention.

Of note, hypothermia occurred at similar rates across all months in the present study and thus represents an important mortality risk factor year-round. Prevention of hypothermia in the prehospital setting, however, has recently gained more attention. The third edition of the Guidelines for Prehospital Management of Traumatic Brain Injury specifically recommends prehospital temperature measurement and efforts to maintain temperatures at 36–37 °C [23]. Future studies should examine whether increased temperature measurement increases prehospital hypothermia management and whether this improves functional and mortality outcomes in pediatric TBI patients.

### Limitations

The findings of the present study should be considered in light of certain data limitations. First, the measurement of patient outcomes was assessed based on hospital discharge status. Due to the high number of in-hospital deaths for hypothermic patients, results indicating hypothermic patients were discharged to inpatient rehabilitation less often were likely influenced by survival bias. Ideally, a more granular measure, such as the Glasgow Outcome Scale – Extended, Pediatric Revision (GOS-E Peds), could be used to assess patient outcomes [24]. However, these data were unavailable for this cohort, so discharge status was used as a proxy. The mortality findings should be interpreted as unadjusted associations. The relatively small cohort and number of outcome events, together with substantial overlap among clinically relevant covariates, limited our ability to reliably adjust for injury severity and other potential confounders. Consequently, the observed association between hypothermia and mortality may reflect greater overall injury severity rather than an independent effect of hypothermia. Although all patients sustained TBI, cause-of-death data were unavailable, and deaths may have resulted from the brain injury, associated extracranial injuries, subsequent complications, or a combination of factors.

This was a retrospective study conducted at a single pediatric trauma center using data collected from 2015 to 2018. Prehospital protocols, thermal management practices, and temperature documentation may have changed since the study period, limiting extrapolation of our findings to contemporary EMS practice. Warming interventions during transport may be documented in patient narratives even if the temperature was not reported. Distinguishing between recognition, treatment, and preventive measures of hypothermia is a limitation of the study.

Additionally, the frequency at which patient temperature was not collected during prehospital ground transport makes it difficult to determine how often these patients experience hypothermia, as they likely differ in injury severity and hypothermia risk from patients transported by air. It is possible that patients with better thermoregulation were less likely to have temperatures recorded. Although the majority of patients with missing temperature measurements arrived at the ED normothermic, almost one-third were hypothermic, and 15% had temperatures below 34 degrees. We kept all cases in the analyses and used all available data, coding temperature as “not collected” after an extensive chart review for every stage of care.

## Conclusion

This study contributes to the growing literature on prehospital risk factors for mortality in pediatric TBI patients. Episodes of hypothermia during early care transitions in pediatric TBI patients were associated with increased in-hospital mortality and may represent targets for intervention before arrival at the trauma center. Future studies should examine the impact of prehospital temperature management on outcomes and mortality in pediatric TBI patients.

## Data Availability

The data that support the findings of this study were obtained from the electronic health record system at Riley Hospital for Children at Indiana University Health and are not publicly available due to patient privacy and institutional restrictions.

## Declarations

### Generative AI declaration statement

The authors did not use a generative artificial intelligence (AI) tool or service to assist with the preparation or editing of this work. The author(s) take full responsibility for the content of this publication.
